# Gambiense Human African Trypanosomiasis Sequelae after Treatment: A Follow-Up Study 12 Years after Treatment

**DOI:** 10.3390/tropicalmed5010010

**Published:** 2020-01-11

**Authors:** Junior Mudji, Anna Blum, Leticia Grize, Rahel Wampfler, Marie-Thérèse Ruf, Lieselotte Cnops, Beatrice Nickel, Christian Burri, Johannes Blum

**Affiliations:** 1 Hôpital Evangélique de Vanga, Vanga Mission, B.P. 4728 Kinshasa 2, Democratic Republic of the Congo; 2 Department of family medicine and primary care, Protestant University of Congo, B.P. 4745, Kinshasa 2, Democratic Republic of the Congo; 3 Swiss Tropical and Public Health Institute, 4002 Basel, Switzerland; 4 University of Basel, 4001 Basel, Switzerland; 5 Institute of Tropical Medicine, 2000 Antwerp, Belgium

**Keywords:** human African trypanosomiasis, sequelae, serology, treatment, oligosymptomatic HAT

## Abstract

The clinical presentation of Human African Trypanosomiasis (HAT) due to *Trypanosoma brucei gambiense* is well known, but knowledge on long-term sequelae is limited. In the frame of studies conducted between 2004 and 2005 in the Democratic Republic of the Congo (DRC), the prevalence of HAT related signs and symptoms were evaluated before the start of treatment and at the end of treatment. To explore possible long-term sequelae, the same clinical parameters were assessed in 2017 in 51 first stage and 18 second stage HAT patients. Signs and symptoms 12–13 years after treatment were compared to before and immediately after treatment and to controls matched for sex and age (±5 years). In first stage HAT patients, the prevalence of all signs and symptoms decreased compared to before treatment but were still higher after 12–13 years than immediately at the end of treatment and in the control group. In second stage HAT patients, all HAT-specific findings had continuously decreased to the point where they were in the range of the healthy control group. In a selection of oligosymptomatic first stage HAT patients, no trypanosomes were detected in the blood by microscopic examination or PCR. An oligosymptomatic presentation of HAT due to the persistence of parasites in compartments, where first stage HAT medications do not penetrate, could not be ruled out.

## 1. Introduction

Human African trypanosomiasis (HAT) is caused by the protozoan parasites *Trypanosoma brucei gambiense (T.b. gambiense)* and *Trypanosoma brucei rhodesiense (T.b. rhodesiense*), which are transmitted by tsetse flies. Whereas the *T.b. gambiense* form is characterized by a progressive course typically lasting three years [1], the *T.b. rhodesiense* form is usually acute, and death occurs within weeks or months of infection.

*T.b. gambiense* is endemic in foci in Western and Central Africa and today causes more than 98% of reported cases of HAT. The disease occurs in two stages, the first, or hemolymphatic, stage without invasion of the central nervous system (CNS) and the second, or neurological, stage with invasion of the CNS by the trypanosomes.

According to the last WHO report (WHO interim guidelines for treatment of gambiense human African trypanosomiasis, August 2019) [2], the worldwide number of *T.b. gambiense* HAT cases dropped from over 25,000 in the year 2000 to below 1000 reported cases worldwide in 2018 [2].

Fever, headache, pruritus, lymphadenopathy, and, to a lesser extent, hepato-splenomegaly are the leading signs and symptoms of the first stage but may also be present, to a lesser degree, in the second stage. During the second stage, neuro-psychiatric disorders such as lethargy, aggressive behaviour, logorrhoea, psychotic reactions, mood changes, and sleep disturbances/disorders dominate the clinical presentation. The neurological symptoms include tremor, general motor weakness, paralysis of an extremity, epilepsy, akinesia, and abnormal movements (dyskinesia, unspecific movement disorders, Parkinson-like movements, speech disorders) [3,4,5,6,7,8]. Sleep disorder with somnolence and short interposed sleeping episodes during the day and at night are imposing clinical symptoms from which “sleeping sickness” derives its name. Total sleep duration, however, remains normal [9].

HAT had always been perceived and described as inevitably fatal if untreated. However, oligosymptomatic forms of HAT with few symptoms, non-detectible parasites, and persistent serological titers were recently described along with their potential role for transmission of HAT [10,11]. The clinical presentation of *T.b. gambiense* HAT has been well documented, but studies on long-term sequelae of HAT have not been performed. The present observational case control study describes the clinical signs and symptoms of HAT patients before treatment and 12–13 years after.

## 2. Materials and Methods

### 2.1. Study Design and Setting (See also Flowcharts below)

The present study assessed the prevalence of HAT related long-term clinical sequelae (signs and symptoms 12–13 years after treatment) and compared signs and symptoms of the HAT patients before, immediately after, and 12–13 years after treatment. Patients at follow-up time were also compared with controls matched by sex and age (±5 years).

This follow-up study was conducted in two phases from 19 July to 14 September 2017 and from 3 May to 30 May 2019 at the Hôpital Evangélique de Vanga, located in the Kwilu province of the Democratic Republic of the Congo (DRC). The area is rural; villages are very remote and only accessible with major efforts by very difficult roads.

### 2.2. Participants

In a clinical study carried out in 2004 on endocrinological changes and the involvement of the heart in second stage HAT (detection of parasite, pathological cerebrospinal fluid), clinical parameters from 29 patients were assessed before treatment, at the end of treatment, and after a follow-up of three months [12,13,14]. Additionally, in the framework of clinical trials carried out between 2004 and 2005, clinical parameters were documented before and after treatment (but without a follow-up of three months) in 96 first stage HAT patients (parasitology confirmed) in Vanga. In these trials, the safety and efficacy of the newly developed drug pafuramidine was compared to the standard treatment with pentamidine for first stage HAT [15,16].

A list of all formerly recruited patients in the above-mentioned studies was compiled, and their place of residence (village) was traced. The villages for the recruitment of study participants were chosen according to the number of eligible patients living there and the accessibility of the villages (distance to Vanga hospital, navigability of roads, and distance between affected villages). According to these parameters, visits were planned, starting with a visit that allowed the enrollment of the largest possible number of patients. Accessible persons were included in the study after informed consent with the exception of patients with one of the following exclusion criteria: history of severe chronic diseases such as tuberculosis, HIV, cancer, liver cirrhosis, and diabetes mellitus. In patients who died or were not accessible, a third-party history by a family member or friend was taken. These patients were not included in the analysis but were discussed separately.

### 2.3. Sample Size

Different clinical parameters of HAT have largely different frequencies. The calculation of a sample size yielding 80% power (α = 0.05) was, e.g., 11 patients for sleeping disorders versus 485 patients for abnormal behavior. It was therefore decided to aim at a minimum 60 patients, which would be sufficient to allow the covering of the major signs and symptoms. Due to the limited number of available patients, the sample size calculation was only performed for the first stage HAT patients. Ninety-six first stage and 29 second stage patients who participated in the former studies lived in the perimeter of the Vanga hospital.

### 2.4. Study Procedure/Clinical Examination

The study was conducted in two parts—a site visit to the villages as described above, followed by a laboratory assessment at the hospital for the first stage HAT patients with clinical signs and symptoms (see below). The original working hypothesis was that no or only rare sequelae or signs of continued infection would be found in treated HAT patients revisited more than 10 years after treatment. Therefore, the study focused on clinical signs and symptoms of HAT patients 12–13 years after treatment. The second part of the study emerged from the findings, which had revealed a significant number of first stage HAT patients with continued or returning complaints. The resulting follow-up study, comprising blood examinations, could only be done with about two years of delay, due to the necessary protocol amendment, time-consuming ethical clearance, and challenges of accessibility and free movement in the election period in the DRC.

For each former patient (=a case), a control person matched by age (±5 years) and sex was enrolled. Family members were preferred and, if not available, hospital personnel or patients with minor surgical problems (i.e., herniotomy) were enrolled instead.

In all HAT patients and controls, a short history and physical examination was established. Questions regarding the symptoms of HAT were asked in the same manner as in the former original study and trials, and the case report form used to compare signs and symptoms was based on those used in the previous trials. The data collected were compared to those before treatment and after treatment from the former studies.

### 2.5. Additional Blood Examination on Patients with Symptoms

For the second part of the study, 15 first stage HAT patients who were still oligosymptomatic and had shown clinical signs or symptoms such as headache, sleep disorders, pruritus, or minor neurological problems during the village visits were invited to report to the hospital for additional tests. The following laboratory tests were performed: Microscopic examination of the blood and the aspiration fluid of enlarged lymphatic glands (if present) for the presence of trypanosomes, serologic testing for trypanosomiasis (CATT/*T.b.gambiense*, Institute of Tropical Medicine, Antwerp, Belgium;IFA, in-house test, Swiss Tropical and Public Health Institute, Basel, Switzerland), and PCR from blood samples. CATT was performed immediately after blood withdrawal in the DRC, and EDTA-blood and serum samples for IFA and PCR were stored in aliquots at −20 °C at the study site until transfer on dry ice to Switzerland.

### 2.6. T.b. gambiense Card Agglutination Test for Trypanosomiasis (CATT)

The CATT is a direct agglutination test using lyophilized bloodstream forms of *T.b. gambiense* variable antigen type LiTat 1.3. It detects specific antibodies in the blood, serum, or plasma of patients infected with *T.b. gambiense* [17]. The test was performed according to the kit manual. For each participant, one CATT test was performed on undiluted whole blood.

The CATT exhibits a sensitivity of 87%–98% [17,18] and a specificity of 95.9% on undiluted whole blood. Cross reactivity with antibodies generated against *Plasmodium* spp. and other parasites is possible.

### 2.7. Trypanosoma brucei IFA, Malaria IFA, and Leishmania IFA Serological Tests

Immunofluorescence staining was performed with bloodstream forms of *T.b. brucei* (strain STIB 345) in addition with *Plasmodium falciparum* (strain NF54) or liver sections of *Leishmania donovani* (strain MHOM-ET-67/L82) infected hamster mounted on glass slides. Slides were stored at −80 °C until day of use. For IFA analysis, slides were quickly air-dried at room temperature and fixed with acetone. Sera were diluted in PBS pH 7.2 and applied to the slide-slots. Three control sera were present on each slide; one positive, one equivocal, and one negative control. After 25 min of incubation at 37 °C in a wet chamber, slides were washed with PBS pH 7.2 and air-dried. FITC conjugated F(ab)’2 anti-IgG/A/M (BioRad, #30244) diluted in 0.01% Evans blue in PBS was added, slides were incubated for 25 min at 37 °C, washed, dried, and a cover glass was mounted with buffered glycerol. Slides were examined immediately with a fluorescence microscope. The serodiagnostic test exhibits a sensitivity of 98% for infections with *T.b. rhodesiense* or *T.b. gambiense* and a specificity of >99% for serum samples from healthy blood donors. The IFA exhibits a sensitivity of >99% for *Plasmodium* spp. and a specificity of 98% for healthy blood donor samples. The test has a sensitivity of >96% for visceral leishmaniasis and a specificity of >99% for heathy blood donor sera. Cross reactivity between the mentioned parasites antigens is possible.

### 2.8. Plasmodium falciparum spp. ELISA

Malaria serology was performed with an in-house screening ELISA for detection of specific *Plasmodium* spp. antibodies. *P. falciparum antigen* (strain NF54) was coated in 0.05 M sodium carbonate buffer, pH 9.6, to Immulon 2HB plates (, Thermo Scientific, Wohlen, Switzerland,). After washing, diluted sera were added to the plates and incubated for 15 min at 37 °C. After additional washing steps, horseradish peroxidase conjugated goat-anti-human-IgG (KPL, 474-1006, BioConcept Ltd, Allschwil, Switzerland) was added. Plates were incubated for 15 min at 37 °C, subsequently washed, and o-Phenylendiamine Dihydrochloride (OPD, Sigma, Buchs, Switzerland) was added. Reaction was stopped with 8 M H_2_SO_4_, and absorption was read with a Multiscan FC reader (ThermoScientific, Wohlen, Switzerland) at 492 nm. All sera giving positive or equivocal results were additionally tested with an in-house confirmatory Malaria IFA.

### 2.9. Real-Time PCR

For molecular analysis, 5 mL of whole blood samples were lysed and stored at the hospital site by adding an equal volume of guanidine-HCl 6 M with 0.1 M EDTA pH = 8, according to previous publications [19,20]. These samples were kept at 4 °C until shipment to Switzerland. Upon arrival, samples were immediately processed. For this, samples were heated at 100 °C for 15 min, and 500 µL of the guanidine-HCl-EDTA whole blood was used for extraction using the QIAamp DNA Mini Kit from Qiagen (Hilden, Germany), according to the manufacturer’s instructions. DNA was eluted in 100 µL AE buffer (provided by the kit). The PCR targets the 18S rRNA gene of the *Trypanozoon* spp. (including the detection of *T.b. brucei, T.b. gambiense, T.b. rhodesiense, T. evansi*, and *T. equiperdum*) as previously described for a conventional PCR format [21]. Primer and probe sequences were newly designed and adapted to the real-time PCR format (forward primer: 5′-TAGTTTTGTGCCGTGCCAGT-3′, reverse primer: 5′-CGCTCCCGTGTTTCTTGTATC-3′, and TaqMan probe: 5′-FAM-TCGGACGTG-iQ500-TTTTGACCCACGC-BHQ1-3′), resulting in an amplicon of 97 basepairs. The PCR reaction mixture contained 5 µL DNA, 1x GoTaq Probe Master Mix from Promega (Madison, USA), 800 nmol forward and reverse primer, and 200 nmol probe in a total reaction volume of 25 µL. The PCR program started with a step at 50 °C for 2 min and 95 °C for 10 min, followed by 45 cycles of 95 °C for 15 s and 58 °C for 1 min. Plasmid containing the 97 bp amplicon sequence, as well as adjacent base pairs, was used as a positive control. The assay was optimised on *T.b. gambiense* and *T.b. rhodesiense* culture samples and spiked blood samples. Analytical sensitivity of the assay was 1 plasmid copy/µL DNA, and 5–50 parasites/mL spiked whole blood. Specificity was tested against DNA samples from *Babesia divergensis*, *Cryptosporidium* spp., *Cyclospora cayetanensis*, *Leishmania aethiopica*, *L. braziliensis*, *L. donovani*, *L. infantum*, *L. mexicana*, *Neospora caninum*, *Plasmodium falciparum*, *P. vivax*, *P. knowlesi*, *P. malariae*, *P. ovale*, *Sarcoycstis hominis*, *Toxoplasma gondii*, and *Trypanosoma cruzi,* and were found to be 100%.

### 2.10. Statistical Analysis

Categorical characteristics were summarized as counts and proportions, continuous characteristics as means, standard deviations, medians, and interquartile ranges. Paired comparisons were tested using McNemars’ exact test or the Wilcoxon signed rank test depending on the nature of the compared factors. SAS version 9.4 (2002–2012, SAS Institute, Cary, NC, USA) was used to perform the statistical analysis. The level of significance was set to an alpha level <0.05.

### 2.11. Ethics Statement

The study protocol was approved by Ethikkommission Nordwest-und Zentralschweiz (EKNZ) (2017-00471; 12.4.2017) and Comité d’Éthique de l’Université Protestante au Congo (UPC) (CEUPC) (0044; 13.6.2017). Prior to enrolment, all participants gave informed written consent.

## 3. Results

In the region of Vanga, 96 first stage and 29 second stage HAT patients were recruited into several studies and trials between 2004 and 2005 [12,13,14,15,16]. The recruitment of participants for the present study took place during three visits to the following villages: (i) Kikongo Tango-Milundu, (ii) Nsalu, and (iii) Mayoko-Nkai.

**Figure 1:**
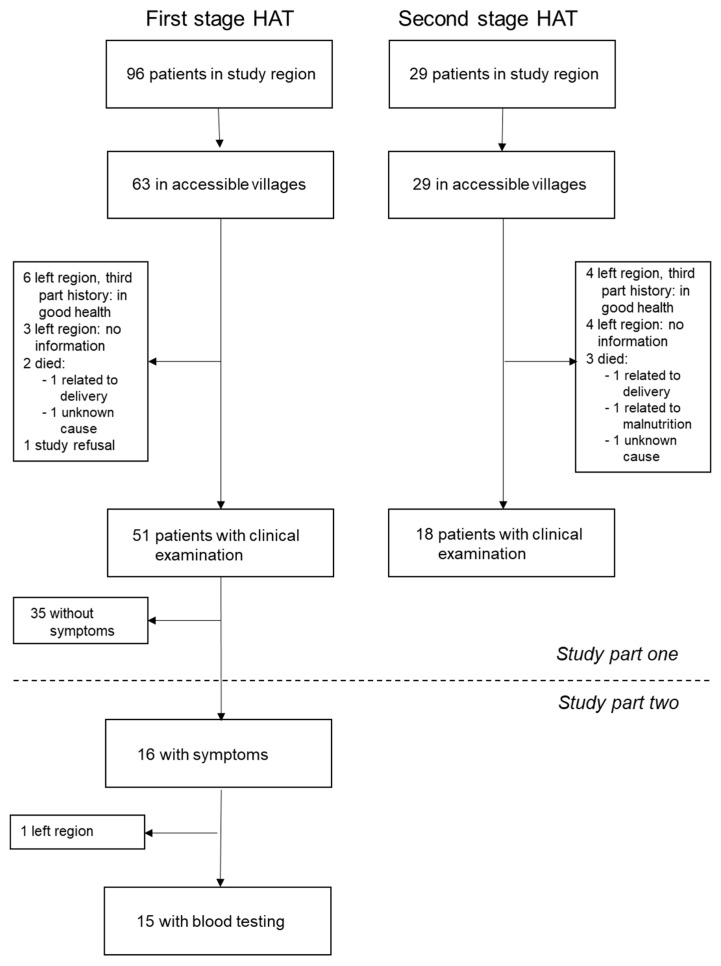
Flow chart of the Human African Trypanosomiasis (HAT) study.

The flowchart of the study, including the number of participants, is shown in Figure 1.

In 69 out of 92 (75%) of the eligible patients, a history and clinical examination could be performed. Fifty-one were previous first stage and 18 were previous second stage HAT patients. Ten out of 92 (11%) were not accessible but, according to oral communication by relatives or friends, were all in good health without any long-term sequelae of HAT. Five out of 92 (5%) died, two of them in the peripartum period of causes unrelated to HAT, and three without a clear diagnosis. Of these three patients, one died due to malnutrition, probably unrelated to HAT, one due to an undefined disease with unspecified swelling but without fever, and the third one due to unknown causes one month after HAT treatment. No information was available for eight out of 92 (9%) of the former patients. There was no history of a confirmed relapse for any of the patients followed up in this study. During the village visits, only clinical investigations, but no specific HAT diagnostic tests, were performed. At this point, no patient was suspected to have HAT; therefore, no blood tests had been planned for this phase of the study.

**Figure 2:**
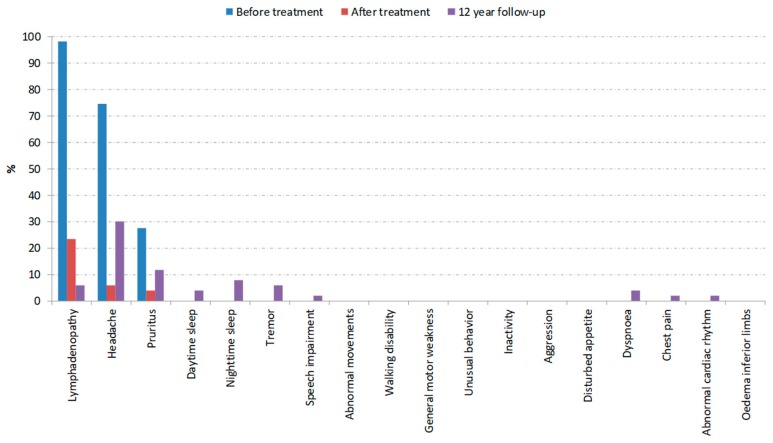
First stage HAT patients: Prevalence of signs and symptoms at different time points. Absence of a bar indicates 0% prevalence at a certain time point, except for dyspnea, chest pain, abnormal cardiac rhythm, and oedema, which were determined only at 12 year follow-up.

**Figure 3:**
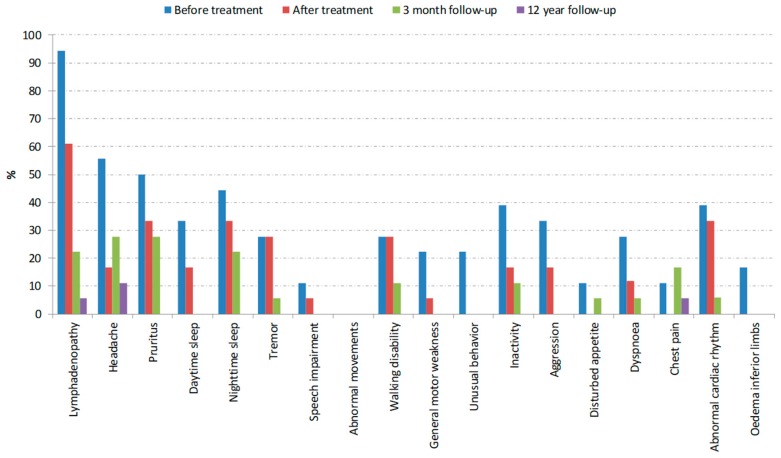
Second stage HAT patients: Prevalence of signs and symptoms at different time points. Absence of a bar indicates 0% prevalence at a certain time point.

Table 1 shows the characteristics for patients with previously first (n = 51) and second (n = 18) stage HAT and their matched controls. Table 2 and Table 3 show the prevalence of signs and symptoms before treatment and at the long-term follow-up, for first and second stage HAT, respectively. Figure 2 and Figure 3 show the development of these signs and symptoms at the different examined time points.

### 3.1. Lymphadenopathy

Lymphadenopathy was the most frequently observed sign before treatment in first stage (98%) and second stage (94.4%) HAT patients. Twelve years after treatment, it was detected only in 5.9% first and in 5.6% second stage patients, which was comparable to the control groups (3.9% and 0%).

### 3.2. Headache

Headache was the most frequent complaint of HAT patients before treatment and was more frequent in the first stage (74%, including 2% with unbearable headache) than in the second stage (55.6%). In first stage HAT, headaches decreased to 6% at the end of treatment but increased thereafter to 30% (none with unbearable headache) 13 years later, which is significantly higher than in the control group (14%). One patient asked for further examination pertaining to the headache, which revealed the cause of the headache to be posttraumatic after a head injury without association to HAT. In second stage HAT, headaches decreased to 11% 12–13 years after treatment, compared to 5.6% in the control group.

### 3.3. Pruritus

Pruritus was the second most frequent complaint of HAT patients and was less frequent in the first stage (27.4%, including 6% with scratching marks) than in the second stage (50%) before treatment. It decreased to 4% after treatment and re-increased to 11.8% at the 12 year follow-up (none with scratching marks) in first stage and decreased to none in second stage HAT, comparable to the control group (2%).

### 3.4. Sleep Disorder

In first stage HAT, sleep disorders were neither observed before nor after treatment, but 3.9% reported daytime sleep and 7.8% reported sleep disorders during the night 12–13 years later. In second stage HAT, daytime sleep decreased from 33% and night time sleep disorders from 44% before treatment stepwise to none 12 years after treatment, equal to the healthy control group.

### 3.5. Neurological Disorders

In first stage HAT, neurological disorders were not described, before or after treatment, but tremor was reported in 6% and speech disorders in 3% at follow-up. In the second stage, neurological disorders such as speech impairment (11%), walking disability (28%), motor weakness (22%), and unusual behavior (22%) were observed before treatment, decreasing continuously post treatment, and had disappeared completely after 12–13 years.

### 3.6. Blood Analysis4

Blood analysis was performed only in the second phase of the study for symptomatic follow-up patients from the group with previous first stage HAT. Trypanosomes could not be detected in any of these 15 patients, neither by direct blood examination nor by real time PCR. The CATT was negative for all patients, but IFA using *T. b. brucei* antigen was positive in most patients (see Table 4). None of the 15 patients showed a positive *Leishmania* serology. By contrast, all patients exhibited a positive malaria serology in ELISA and IFA, which can be explained by the region being an endemic area for malaria.

## 4. Discussion

To the best of our knowledge, this is the first study assessing the long-term sequelae of first and second stage HAT patients more than 10 years after initial treatment, comparing them to healthy controls. The study evaluated the clinical signs and symptoms before treatment, immediately after treatment, and 12–13 years after treatment in 51 first stage and 18 second stage HAT patients. A surprisingly high percentage (75%) of the eligible and accessible patients could be enrolled in the present study, while information on the health condition (including death) of 15% of the patients could be gathered from relatives and friends. In only 10% of the eligible patients was no information available.

Five cases were lost to follow-up because of death. In two of these, HAT could be ruled out as the cause of death, and in the other three, HAT was unlikely as the cause of death according to information from friends and relatives.

In first stage HAT patients, the prevalence of all signs and symptoms (except lymphadenopathy) had decreased 12–13 years after treatment compared to before treatment, but was still higher than immediately after the end of treatment and in the control group. The high prevalence of headaches (31%), pruritus (12%), and even the presence of neuropsychiatric symptoms such as tremor (6%), speech impairment (2%), and sleeping problems (12%) is worrying. All patients with neuropsychiatric symptoms also suffered from headaches. No relapse was clinically suspected in any of the patients or reported by the patients in the time before the long-term follow-up.

In second stage HAT patients treated with melarsoprol, all HAT specific signs and symptoms decreased steadily at the examined time-points towards a range of the healthy control group 12–13 years post-treatment. Only headache was reported in 11%, but this was not significantly different compared to the control group.

The higher prevalence of symptoms in first stage than in second stage HAT patients at follow-up is surprising and contradictory to former studies. In children treated with melarsoprol, signs and symptoms one month to four years after treatment were more frequently reported in second stage patients (22 symptoms in 28 patients) than in their first stage counterparts (25 symptoms in 92 patients) [22]. In contrast to our results, neither headache nor pruritus were the main symptoms, but were primarily neuropsychiatric symptoms: mental confusion (7.5%), changes in character (11.5%), memory problems (11.5%), language problem (3%), and sleeping disorders (5%). Furthermore, changes in growth in young participants, 6–20 years of age, formerly treated for HAT, were more accentuated in second stage HAT compared to first stage HAT [23]. The subjects treated for first and second stage HAT had a smaller body weight (−4.25 kg), were 3 cm shorter, and had a smaller mid-upper-arm circumference (−1.15 cm) than the control group matched for age, sex and residence. These changes were more accentuated in second stage than in first stage patients: body weight from −4.9 to −2.6 kg, height from −4.9 to −2 cm, and mid-upper-arm circumference from −2 to −1 cm [23]. Differences to these studies could eventually be explained by a relatively low number of patients and a different study design (i.e., age of patients, different follow-up period, different context).

In first stage HAT patients, the clear improvement of the signs and symptoms after treatment was expected, but the reappearance of such symptoms 12–13 years post-treatment is surprising. In a follow-up examination of the patients, no trypanosomes could be detected in any of them, neither by direct microscopic examination nor by real time PCR of the blood. The CATT was negative for all 15 former first stage HAT patients with persistent symptoms, but 80% of them were positive in the *Trypanosoma* serology by IFA. In addition, 13% of the 15 participants showed an equivocal result in IFA, and only 7% were negative. The positive *Trypanosoma* serology by IFA could be explained by either long term-persisting anti-trypanosomal antibodies, a boost of immunity without an actual measurable infection due to repetitive infected tsetse bites [24], the presence of subclinical infection, or by cross-reactivity of malaria antibodies to a certain extent.

There are several hypotheses to explain these unexpected results:

First, the patients improved after the treatment and subjectively did not notice remaining minor symptoms at the end of treatment. All these symptoms were generally mild, and the patients did not seek medical help. It could be possible that the symptoms did not really disappear but persisted at a level below immediate perception. This would mean that sequelae persist even more than a decade after treatment and include headaches, pruritus, sleeping disorders, and rare neurological deficits in the absence of persisting parasites. The description of the signs and symptoms described in children [22,23] and case series of adults [10] strengthens the hypothesis of persistence of sequelae.

Secondly, after an initial cure, some patients could have had a relapse or a reinfection with HAT with an oligosymptomatic presentation and trypanosomes below the detection level in the blood. Even if HAT infections are rare events, mainly in the last decade [25], the study design does not allow the exclusion of such reinfection or relapse.

The third hypothesis could be that a few parasites have survived (hiding in other compartments than the blood) and may have caused a persisting oligosymptomatic low-level infection. Treatment with melarsoprol, which reaches nearly all compartments of the body, killed the trypanosomes, but neither pentamidine nor pafuramidine, used against first stage HAT, can penetrate the blood brain barrier and may not have reached all body compartments. Tanowitz described replicating trypanosomes in adipose tissue interacting with adipocytes [26]. Since most drugs used for the treatment of HAT are hydrophilic, their efficacy could be limited in a lipophilic environment, and adipose tissue could represent a safe site for trypanosomes to hide [26]. It has been described that the skin or the CSF could also represent an anatomic reservoir of trypanosomes [21,27]. In addition, the persistence of trypanosomes in the blood below the detection level cannot be ruled out.

Of interest are the results of a study where untreated HAT patients were compared to formerly treated HAT patients [10]. Eleven HAT patients refused treatment, survived, and were evaluated 2–9 years later. Parasites were detected in only one out of 11 patients in the blood (by microscopy and PCR), and HAT specific antibody titres had decreased in only seven out of 10 patients. The study suggests that oligo-or asymptomatic HAT infections may show spontaneous cure over time or persist as low level infection without passage to the CNS [10]. The control group included 18 first stage and 21 second stage HAT patients treated 15 years prior. No parasites could be detected in the blood of any patients, neither by PCR nor by microscopy after mini anion exchange centrifugation technique. A combination of a positive immune trypanolysis test [28] with a negative CATT test was found in 61.5% [10]. In 58% of the treated and in 68% of the untreated patients, non-specific symptoms such as fever and headaches were observed, showing that such symptoms may persist for a long time [10].

The findings lead to the hypothesis that a few surviving trypanosomes in certain compartments could partially be controlled by the immune system, leading to minor persisting symptoms without, however, the development of apparent clinical signs of a relapse of the disease. The persistent survival of trypanosomes could be a source of transmission [10,11] and could explain the reactivity of the serum in the *Trypanosoma* IFA in our study. The negative CATT results could possibly be explained by either a lower sensitivity than the IFA, by the occurrence of trypanosome strains that lack or do not express the LiTat 1.3 gene that has been described for some endemic areas in Nigeria [17], or by the rapid decline of CATT reactivity in treated HAT patients within a few years [29] while other serological tests are still positive. On the other hand, the negative CATT results could also mean that anti-trypanosomal antibodies are no longer present and the positive IFA results are caused by cross-reacting malaria antibodies.

A review of the natural progression of HAT concludes that, in rare cases, an oligosymptomatic chronic carrier stage exists, but duration beyond six to seven years is exceptional [30]. It is also worth paying attention to the broad spectrum of clinical presentations of *T.b. gambiense* HAT, ranging from a chronic oligosymptomatic form to the frequently observed, usually fatal chronic disease in endemic populations to the acute clinical presentation in non-endemic populations [31].

The presented results would be compatible with such a hypothesis, and the presence of antibody titres even 14–15 years after the end of treatment supports this hypothesis, although it needs to be confirmed by more robust data. The study demonstrates a surprisingly high percentage of HAT patients still suffering from symptoms even more than 10 years after treatment. The study was not designed to specify if re-infections, relapses, or parasites surviving in not yet well described body compartments cause the signs and symptoms or if the patients have sequelae without any surviving trypanosomes. These questions should be addressed in further studies.

## 5. Limitations

The limitations of this study are the subjective character of the symptoms, the difficulties associated with properly assessing third party history, and the relatively low number of patients. Another limitation of the study is that no *Trypanosoma* real time PCR and no *Trypanosoma* and malaria serology was performed for treated asymptomatic patients or control group participants. These data could not be collected due to the study design and the unexpected outcome of the first phase of the study. Finally, cross-reactivity of malaria antibodies against *T. brucei* antigens or vice versa cannot be ruled out.

## 6. Conclusions

We demonstrated HAT-related signs and symptoms more than 10 years after treatment for the first time in first stage HAT patients without detectable levels of parasites. In first stage HAT patients, the prevalence of all signs and symptoms decreased compared to before treatment, but were still higher than at the end of treatment and in the control group. In second stage HAT patients, all HAT specific findings had continuously decreased to the point where they were in the range of the healthy control group. Persistence of symptoms without detectable levels of parasites in the blood could suggest either parasitaemia below the detection level or the persistence of parasites in some other body compartments. Further studies, including skin or tissue biopsies for detection of trypanosomes by PCR and serologic testing before treatment as well as ten years later, could indicate if there is a reservoir, which could be relevant for subclinical forms and transmission.

## Figures and Tables

**Table 1:** Descriptive characteristics for first and second stage HAT patients and their controls.

Characteristic	First Stage HAT		Second Stage HAT	
	Patients (n = 51)	Controls (n = 51)	Patients (n = 18)	Controls (n = 18)
Age * (years):				
Mean ± SD	43.9 ± 12.5	42.7 ± 12.5	52.0 ± 15.8	50.9 ± 14.5
Median (IQR)	41 (35 to 50)	42 (32 to 51)	47.5 (37 to 64)	48 (36 to 64)
Gender: n (%)				
Male	14 (27.5)	14 (27.5)	9 (50.0)	9 (50.0)
Female	37 (72.5)	37 (72.5)	9 (50.0)	9 (50.0)

* Age of controls matched within ±5 years of age of patients; SD = standard deviation; IQR = interquartile range (first to third quartile).

**Table 2:** First stage HAT: Signs and symptoms for patients before treatment and at 12 year follow-up and for their controls.

Symptom/Sign	Level	Before Treatment	12 Year Follow-up	*p*-Value *	Controls ^†^	*p*-Value *,^§^
		n (%)	n (%)		n (%)	
Lymphadenopathy	Absent	1 (2.0)	48 (94.1)	<0.0001	49 (96.1)	0.6547
	Palpable	50 (98.0)	3 (5.9)		2 (3.9)	
Headache	Absent	13 (26.0)	35 (70.0)	<0.0001	43 (86.0)	0.0325
	Present/Unbearable	37 (74.0)	15 ^(4)^ (30.0)		7 (14.0)	
Pruritus	Absent	37 (72.6)	45 (88.2)	0.0005	50 (98.0)	0.0588
	Present/Visible traces of scratching	14 (27.4)	6 ^(4)^ (11.8)		1 (2.0)	
Daytime sleep	Normal	51 (100.0)	49 (96.1)	-	51 (100.0)	-
	Repeatedly/Continuously	0 (0.0)	2 ^(2)^ (3.9)		0 (0.0)	
Nighttime sleep	Normal	51 (100.0)	47 (92.2)	-	51 (100.0)	-
	Few hours/Rare	0 (0.0)	4 ^(4)^ (7.8)		0 (0.0)	
Tremor	Absent	51 (100.0)	48 (94.1)	-	51 (100.0)	-
	Visible/Severe	0 (0.0)	3 ^(3)^ (5.9)		0 (0.0)	
Speech impairment	Absent	51 (100.0)	50 (98.0)	-	51 (100.0)	-
	Present/Non-interpretable	0 (0.0)	1 ^(1)^ (2.0)		0 (0.0)	
Abnormal movement	Absent	51 (100.0)	51 (100.0)	-	-	-
Walking disability	Absent	51 (100.0)	51 (100.0)	-	-	-
General motor weakness	Absent	51 (100.0)	51 (100.0)	-	-	-
Unusual behavior	Absent	51 (100.0)	51 (100.0)	-	-	-
Inactivity	Absent	51 (100.0)	51 (100.0)	-	-	-
Aggression	Absent	51 (100.0)	51 (100.0)	-	-	-
Disturbed appetite	Normal	51 (100.0)	51 (100.0)	-	-	-

* McNemar’s exact test; ^†^ Each patient was matched to a control by sex and age (±5 years); ^§^ For patients at 12 year follow-up and their healthy controls; ^(x)^ Number of new onsets.

**Table 3:** Second stage HAT: Signs and symptoms for patients before treatment and at 12 year follow-up and for their controls.

Symptom/Sign	Level	Before Treatment	12 Year Follow-Up	*p*-Value *	Controls ^†^	*p*-Value *,^§^
		n (%)	n (%)		n (%)	
Lymphadenopathy	Absent	1 (5.6)	17(94.4)	0.0020	17 (100.0)	-
	Palpable	17 (94.4)	1 (5.6)		0 (0.0)	
Headache	Absent	8 (44.4)	16 (88.9)	0.0156	17 (94.4)	0.5637
	Present/Unbearable	10 (55.6)	2 ^(1)^ (11.1)		1 (5.6)	
Pruritus	Absent	9 (50.0)	18 (100.0)	-	18 (100.0)	-
	Present/Visible traces of scratching	9 (50.0)	0 (0.0)		0 (0.0)	
Daytime sleep	Normal	12 (66.7)	18 (100.0)	-	18 (100.0)	-
	Repeatedly/Continuously	6 (33.3)	0 (0.0)		0 (0.0)	
Nighttime sleep	Normal	10 (55.6)	18 (100.0)	-	18 (100.0)	-
	Few hours/Rare	8 (44.4)	0 (0.0)		0 (0.0)	
Tremor	Absent	13 (72.2)	18 (100.0)	-	18 (100.0)	-
	Visible/Severe	5 (27.8)	0 (0.0)		0 (0.0)	
Speech impairment	Absent	16 (88.9)	18 (100.0)	-	18 (100.0)	-
	Present/Non-interpretable	2 (11.1)	0 (0.0)		0 (0.0)	-
Abnormal movement	Absent	18 (100.0)	18 (100.0)	-	18 (100.0)	-
Walking disability	Absent	13 (72.2)	18 (100.0)	-	18 (100.0)	-
	Present/Walking with help/inability to walk	5 (27.8)	0 (0.0)	-	0 (0.0)	
General motor weakness	Absent	14 (77.8)	18 (100.0)	-	18 (100.0)	-
	Ability to stand from chair no hands/No ability to stand	4 (22.0)	0 (0.0)	-	0 (0.0)	
Unusual behavior	Absent	14 (77.8)	18 (100.0)	-	18 (100.0)	-
	Visible/Severe	4 (22.0)	0 (0.0)	-	0 (0.0)	
Inactivity	Absent	11 (61.1)	18 (100.0)	-	18 (100.0)	-
	Reduced workforce/Inability to perform daily tasks	7 (38.9)	0 (0.0)	-	0 (0.0)	
Aggression	Absent	12 (66.7)	18 (100.0)	-	18 (100.0)	-
	Sporadic/Severe, requires observation	6 (33.3)	0 (0.0)	-	0 (0.0)	
Disturbed appetite	Normal	16 (88.9)	18 (100.0)	-	18 (100.0)	-
	Disturbed/Severely	2 (11.1)	0 (0.0)	-	0 (0.0)	
					

* McNemar’s exact test; ^†^ Each patient was matched to a control by sex and age (±5 years); ^§^ For patients at 12 year follow-up and their healthy controls; ^(x)^ Number of new onsets.

**Table 4:** Real-time PCR, CATT, *T. brucei*, Malaria, and *Leishmania* serology of 15 follow-up patients.

	Real-time PCR Trypanosoma	CATT Test	*T. Brucei* Serology		Malaria Serology			Leishmania Serology	
Serum	Result	Result	IFA (Titre)	Result	ELISA (OD)	IFA (Titre)	Result	IFA (Titre)	Result
P07	negative	negative	1/320	positive	1.59	1/1280	positive	<1/80	negative
P14	negative	negative	1/160	equivocal	1.00	1/1280	positive	<1/80	negative
P28	negative	negative	1/640	positive	1.84	1/1280	positive	<1/80	negative
P29	negative	negative	<1/160	negative	1.62	1/1280	positive	<1/80	negative
P42	negative	negative	1/320	positive	1.95	1/1280	positive	<1/80	negative
P50	negative	negative	1/320	positive	1.82	1/1280	positive	<1/80	negative
P51	negative	negative	1/640	positive	1.76	1/1280	positive	<1/80	negative
P53	negative	negative	1/320	positive	1.65	1/1280	positive	<1/80	negative
P55	negative	negative	1/640	positive	1.71	1/1280	positive	<1/80	negative
P56	negative	negative	1/320	positive	1.73	1/1280	positive	<1/80	negative
P57	negative	negative	1/320	positive	1.30	1/640	positive	<1/80	negative
P58	negative	negative	1/640	positive	1.26	1/1280	positive	<1/80	negative
P62	negative	negative	1/320	positive	1.74	1/1280	positive	<1/80	negative
P63	negative	negative	1/160	equivocal	1.74	1/1280	positive	<1/80	negative
P64	negative	negative	1/320	positive	2.09	1/1280	positive	<1/80	negative

Cutoffs, T. brucei IFA: <1/160 negative, 1/160 equivocal, ≥1/320 positive; Malaria ELISA: <0.15 negative, 0.15–0.29 equivocal, ≥0.30 positive; Malaria IFA: <1/40 negative, 1/40 equivocal, ≥1/80 positive; Leishmania IFA: <1/80 negative, 1/80 equivocal, ≥1/160 positive.
